# Shortwave-infrared (SWIR) fluorescence molecular imaging using indocyanine green–antibody conjugates for the optical diagnostics of cancerous tumours†

**DOI:** 10.1039/d0ra04710d

**Published:** 2020-07-28

**Authors:** Setsuko Tsuboi, Takashi Jin

**Affiliations:** RIKEN Center for Biosystems Dynamics Research (BDR), RIKEN Furuedai 6-2-3, Suita Osaka 565-0874 Japan tjin@riken.jp; Graduate School of Frontier Biosciences, Osaka University Yamada-oka 1-3, Suita Osaka 565-0871 Japan

## Abstract

Recently, shortwave-infrared (SWIR, 1000–1400 nm) fluorescence imaging has attracted much attention due to the higher contrast and sensitivity with deeper penetration depths compared to conventional visible and near-infrared (NIR) fluorescence imaging. For the SWIR fluorescence imaging, the development of fluorescent probes emitting over 1000 nm is necessary. So far, a variety of SWIR fluorescent probes based on single-walled carbon nanotubes, quantum dots, rare-metal doped nanomaterials, and organic dyes have been developed. However, there are a very limited number of biocompatible SWIR fluorescent probes, which can be used to biomedical applications. Among NIR and SWIR fluorescent probes, indocyanine green (ICG) is the only fluorescent dye approved by US Food and Drug Administration (FDA) for clinical use. Although ICG has a fluorescence maximum at a NIR region (*ca.* 830 nm), ICG emits in the SWIR region over 1000 nm. Here, we present ICG-based SWIR fluorescence molecular imaging for the highly-sensitive optical detection of breast and skin tumours in mice. As SWIR fluorescent molecular-imaging probes, we synthesized ICG–antibody conjugates, which prepared from anti-HER2 antibody (Herceptin), anti-EGFR antibody (Erbitux), anti-VEGFR-2 antibody (Cyramza), and anti-PD-L1 antibody (anti-PD-L1 ab). The present SWIR molecular imaging probes specifically accumulated to the breast and skin tumours, and their SWIR fluorescence images (>1000 nm) showed 1.5–2.0 times higher contrast than NIR tumour images taken at 830 nm. We show that the SWIR fluorescence imaging using ICG–antibody conjugates can be used for the elucidation of expression level of cancer-specific membrane proteins, HER2, EGFR, VEGFR-2, and PD-L1 *in vivo*. We also show that the SWIR fluorescence imaging enables quantitative analysis of the change in the size of tumour treated with an anti-cancer drug, Kadcyla. Our findings suggest that the SWIR fluorescence molecular imaging using ICG–antibody conjugates has potential to use for the optical diagnostics of cancerous tumors in medical and clinical fields.

## Abstract 2

We present indocyanine green (ICG)-based shortwave-infrared (SWIR) fluorescence molecular imaging for the highly-sensitive optical detection of breast and skin tumours in mice.

## Graphical abstract

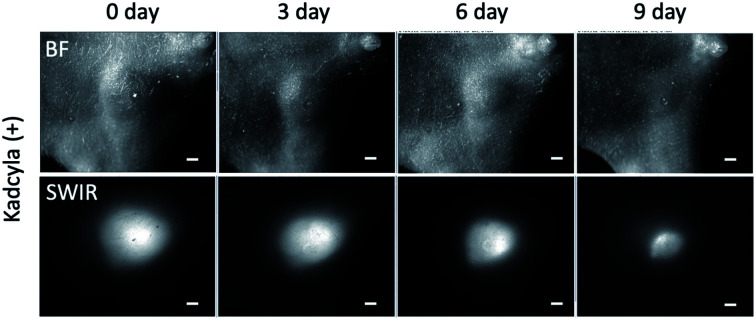

## Introduction

Fluorescence imaging is widely used for the visualization of molecular and cellular structures through labelling with fluorescent probes *in vitro* and *in vivo*.^1^ Recently, fluorescence imaging in the SWIR region of 1000–1400 nm has attracted much attention due to the higher signal to background contrast and sensitivity with deeper penetration depths compared to conventional visible and near-infrared fluorescence imaging.^2^ For the SWIR fluorescence imaging, fluorescent probes emitting over 1000 nm are needed.^2*a*^ So far, several types of SWIR fluorescent probes based on single-walled carbon nanotubes,^3^ quantum dots,^4,5^ rare-metal doped nanomaterials,^6^ and organic-dyes^7^ have been developed.^2*a*^ However, owing to their cytotoxicity, biomedical applications of SWIR fluorescence imaging are limited. At present, there are a few number of SWIR fluorescent probes that can be used to the biomedical and clinical applications.^7*d*,7*l*,7*m*^

In the last few years, organic-dye based fluorescent probes with the low cytotoxicity have been developed for deep-tissue imaging in the SWIR region.^7^ Among the organic-dye based fluorescent probes, indocyanine green (ICG) is the only fluorescent NIR dye approved by US Food and Drug Administration (FDA), which is used in clinical fields.^8^ Although ICG is a traditional NIR fluorescent dye with a fluorescence maximum at *ca.* 830 nm, ICG can emit in the SWIR region.^7*d*,7*l*,7*m*^ ICG is a most popular NIR fluorescent probe for non-invasive deep tissue imaging in animals^9^ and humans.^8^ In the clinical field, ICG is employed for diagnostic investigations of hepatic function^10^ and ophthalmic angiography.^11^ Although ICG is a NIR fluorescent probe emitting at *ca.* 830 nm, recent research has shown that ICG can also be used as a SWIR fluorescent probe for non-invasive deep-tissue imaging over 1000 nm in mice^7*d*,7*l*,7*m*^ and humans.^12,13^ However, there are only a few reports of ICG-based SWIR fluorescence molecular imaging to visualize cancerous tumours in living system.^7*l*,13*a*^

Owing to the lower light scattering and autofluorescence in the SWIR region, SWIR fluorescence molecular imaging using ICG is expected to offer higher signal to background signals compared to conventional visible and near-infrared fluorescence imaging. As ICG-based SWIR molecular imaging probes, we synthesized ICG–antibody conjugates, which are prepared from human epidermal growth factor receptors (EGFR^14^ or HER2 (ref. 15)), and vascular endothelial growth factor receptors (VEGFRs),^16^ and programmed death-ligand 1 (PD-L1).^17^ EGFR and HER2 are transmembrane tyrosine kinase receptors that are overexpressed on the surface of most cancer cells. VEGFRs are signaling proteins which play important roles in the growth of blood vessels in tumours (tumour angiogenesis).^16,18^ PD-L1 is a transmembrane protein that is overexpressed in several types of cancers, where PD-L1 plays a major role to allow cancers to evade the host system.^19^ PD-L1 can also be used as a biomarker for tumour imaging.

As tumour-targeting probes, we used four types of monoclonal antibodies such as anti-HER2 antibody (Herceptin),^20^ anti-EGFR antibody (Erbitux),^21^ anti-VEGFR-2 antibody (Cyramza),^22^ and anti-PD-L1 antibody (anti-PD-L1 ab).^23^ These monoclonal antibodies are used as antibody drugs of cancer therapy for breast and skin tumours in clinical fields. By using the ICG–antibody conjugates, we conducted NIR and SWIR fluorescence imaging of breast and skin tumours in mice. Here, we show that the signal to background contrast in the SWIR tumour imaging is 1.5–2 times better than that of the NIR tumour imaging. The high contrast tumour images in the SWIR region results from the lower tissue absorption/scattering and autofluorescence in the SWIR region. We also show that the high contrast SWIR imaging enables the visualization of the expression level of tumour-specific membrane proteins *in vivo*. Furthermore, we show time-course SWIR fluorescence imaging for tumour shrinking by treatment with an anti-cancer drug, ado-trastuzumab emtansine (Kadcyla).^24^ Our finding suggests that the SWIR fluorescence imaging using ICG–antibody conjugates has potential to apply to the optical diagnostic of cancerous tumours in humans.

## Results and discussion

### Preparation of fluorescent-dye labelled antibody

**Fig. 1:**
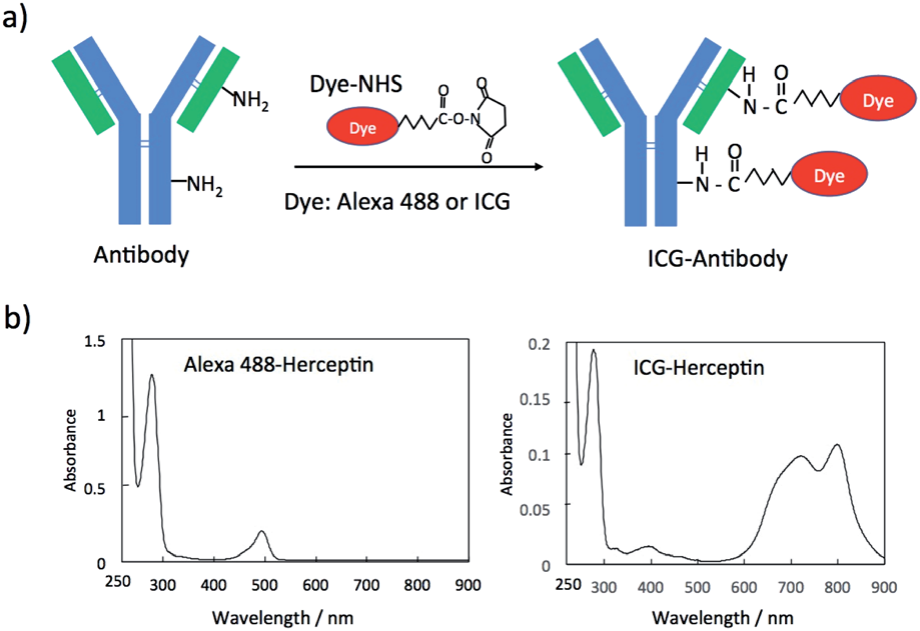
(a) A schematic representation for the conjugation of an *N*-succinimidyl ester derivative (Dye-NHS) of a dye to antibody. Dye-NHS binds to amino groups of a light and heavy chain of an antibody molecule. (b) Absorption spectra of Alexa488 and ICG conjugated Herceptin.

Fluorescent dye and antibody conjugates act as fluorescence molecular imaging probes *in vivo*.^25^ Antibody can be labelled with many types of fluorescent dyes (*e.g.* amine-reactive and sulfhydryl-reactive dyes).^26^ Since there are many monoclonal antibodies for cancer diagnostics and therapy,^27^ we can easily prepare molecular imaging probes by labelling the antibodies with fluorescent molecules. For example, amine-reactive fluorescent dyes such as fluorescein-5-isothiocyanate (FITC) and sulfhydryl-reactive dyes such as fluorescein-5-maleimide bind to amino groups and sulfhydryl groups of antibody molecules, respectively.^26^ In this work, we employed *N*-hydroxysuccinimidyl ester (NHS) derivatives of fluorescent dyes, Alexa488 and ICG for labelling the lysine residues of antibody molecules (Fig. 1a). These NHS derivatives, Alexa488-NHS ester and ICG-NHS ester, easily react with amino groups of antibody molecules at the pH from 7.5 to 9 to form fluorescence labelled antibody molecules. The labelling efficiency of dye to antibody can be controlled by the molar ratio of dye to antibody and the reaction time.

The labelling efficiency was estimated from the absorption spectra of Alexa 488-Herceptin and ICG-Herceptin (Fig. 1b), where the molar extinction coefficients of antibody, Alexa 488, and ICG are 216 000 M^−1^ cm^−1^ (at 280 nm), 71 000 M^−1^ cm^−1^ (at 495 nm), and 147 000 M^−1^ cm^−1^ (at 800 nm), respectively.^28,29^ The labelling efficiency was 50% for Alexa 488-Herceptin and 89% for ICG–Herceptin conjugates. For other antibody conjugates, the labelling efficiencies of the Alexa 488 and ICG dye were summarized in Fig. S1† and S2† together with the absorption spectra of the dye-antibody conjugates. The SDS–PAGE for the ICG–Herceptin conjugate showed that ICG binds to both the light (23 kDa) and heavy chain (49 kDa) of Herceptin (Fig. S3†).

### Expression level of membrane proteins on cancer cells

**Fig. 2:**
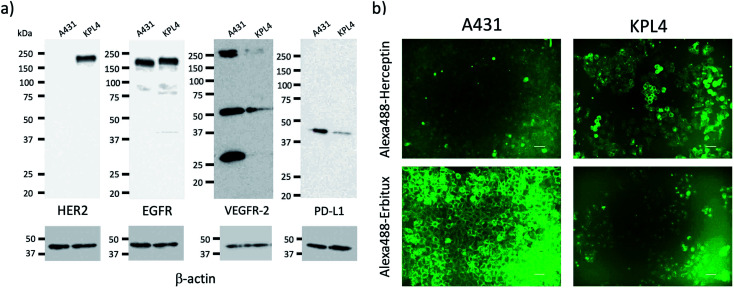
(a) Western blotting analysis for the expression level of HER2, EGFR, VEGFR-2, and PD-L1 in A431 and KPL-4 cells. (b) Fluorescence images of A431 and KPL-4 cells, which were stained with Alexa488–Herceptin and Alexa488–Erbitux. Excitation: 450–490 nm, Emission: 500–550 nm. Scale bar: 50 μm.

For tumour imaging, we prepared two types of cancer model mice, where skin cancer cell (A431)^30^ and breast cancer cell (KPL-4)^31^ were implanted. A431 and KPL-4 cells are the cancer cells established from a human epidermoid carcinoma and a human breast tumour, respectively.^30,31^ To check the expression level of the membrane proteins, HER2, EGFR, VEGFR-2, and PD-L1, we first conducted western blotting analysis for A431 and KPL-4 cells (Fig. 2a). The results show that KPL-4 cells overexpress all the protein, EGFR, HER2, VEGFR-2, and PD-L1, while A431 cells do not overexpress HER2 except for EGFR, VEGFR-2, and PD-L1.

Next, we conducted fluorescence imaging for A431 and KPL-4 cells. For the cellular imaging, we used visible-emitting Alexa488–antibody conjugates instead of ICG–antibody conjugates, because of the low sensitivity of a conventional fluorescence microscope in the NIR region (<700 nm). We prepared Alexa488–antiody conjugates with Herceptin, Erbitux, Cyramza, and anti-PD-L1 ab. Alexa488–Herceptin stained the membrane surface of KPL-4 cell, and its fluorescence was much stronger than that of A431 cells (Fig. 2b). In the case of Alexa488–Erbitux, the membrane surface of both the A431 and KPL-4 cells were stained (Fig. 2b). This observation is consistent with the HER2 and EGFR expression level for the A431 and KPL-4 cells (Fig. 2a). Alexa488–Cyramza and Alexa488–anti-PD-L1 ab also stained both the A431 and KPL-4 cell membranes, although their fluorescence emissions were weaker than those stained by Alexa488–Herception and Alexa488–Erbitux (Fig. S4†). The cellular imaging showed that A431 and KPL-4 cell express the membrane proteins, HER2, EGFR, VEGFR-2, and PD-L1 on their cell surface, although their expression level are different between A431 and KPL-4 cell.

### Fluorescence properties of ICG–antibody conjugates

**Fig. 3:**
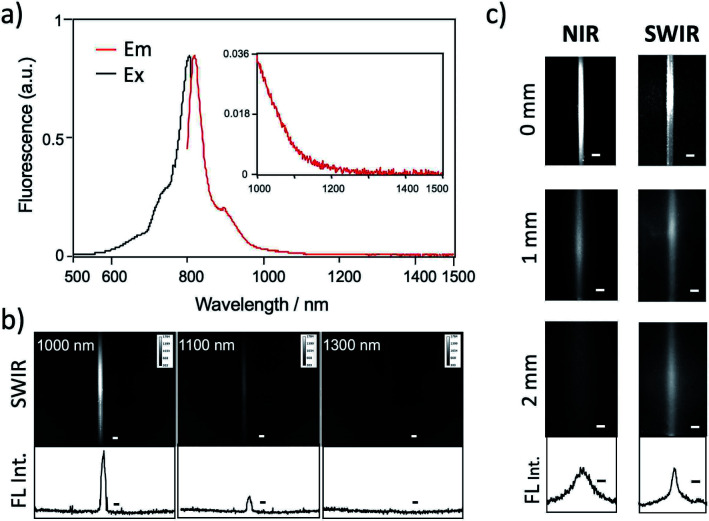
(a) Excitation and emission spectra of ICG–Herceptin. (b) SWIR fluorescence images of a capillary filled with ICG–Herceptin. Fluorescence was observed at 1000, 1100, and 1300 nm. Excitation wavelength was 785 nm. The graphs show the intensity profile for the SWIR fluorescence images. Scale bar: 1 mm. (c) NIR fluorescence (830 nm) and SWIR fluorescence (>1000 nm) images for the capillary filled with ICG–Herceptin, where its fluorescence was passed through an agarose gel (thickness: 0, 1, and 2 mm) containing 1% intralipids. Excitation wavelength was 785 nm. The lower graphs show the intensity profile for the above SWIR image for a 2 mm-agarose gel. Scale bar: 1 mm.

Recent research has proven that ICG fluorescence emission over 1000 nm can be used for the non-invasive deep-tissue imaging of brain and lymph vasculatures.^7*d*,7*l*,7*m*^Fig. 3a shows the excitation and emission spectra of ICG–Herceptin conjugate in an aqueous solution. SWIR fluorescence intensity of ICG–Herceptin over 1000 nm was at least 20 times weak compared to its NIR fluorescence intensity at 830 nm. However, SWIR fluorescence of the ICG–Herceptin has enough intensity to be able to detect by using an InGaAs CCD camera. SWIR fluorescence quantum yield of an ICG–Herceptin conjugate in water was *ca.* 0.5%.^32^ SWIR fluorescence images of a capillary filled with an aqueous solution (2 μM) of ICG–Herceptin are shown in Fig. 3b. The images are taken by using band-path filters (1000 ± 15 nm, 1100 ± 15 nm and 1300 ± 15 nm), where the excitation wavelength of ICG was set to 785 nm. Although SWIR fluorescence intensity of ICG–Herceptin decreased with increasing the detection wavelength, its SWIR fluorescence was detected at the wavelength region from 1000 to 1300 nm.

To check the light scattering in the SWIR region, we detected SWIR fluorescence passed through an agarose gel. Fig. 3c shows the comparison of NIR and SWIR fluorescence emissions of ICG–Herceptin (2 μM). The images were taken for a capillary (0.5 mm in diameter) filled with an aqueous solution of ICG–Herceptin, where its fluorescence was passed through a 1% agarose gel including intralipids (thickness: 0, 1, and 2 mm). The NIR fluorescence images of ICG–Herceptin blurred with increasing the thickness of an agarose gel due to the scattering of the NIR fluorescence. In contrast, the SWIR fluorescence images of ICG–Herceptin were less scattered, resulting in clearer fluorescence images compared to the fluorescence images taken in the NIR region. The full width at half maximum of the SWIR fluorescence image was three-times smaller than that of the NIR fluorescence image (lower graphs in Fig. 3c). The SWIR clearer image results from the lower scattering of SWIR fluorescence compared to NIR fluorescence in the gel.

### High-contrast SWIR fluorescence imaging of breast tumours

Although the SWIR fluorescence of ICG was very weak compared to its NIR fluorescence, higher signal to background contrast of the SWIR fluorescence images was expected due to the lower tissue scattering and autofluorescence in the SWIR region (Fig. 3c and S5†). To compare the image contrast for tumours, we conducted NIR and SWIR tumour imaging using ICG–antibody conjugates in mice.

**Fig. 4:**
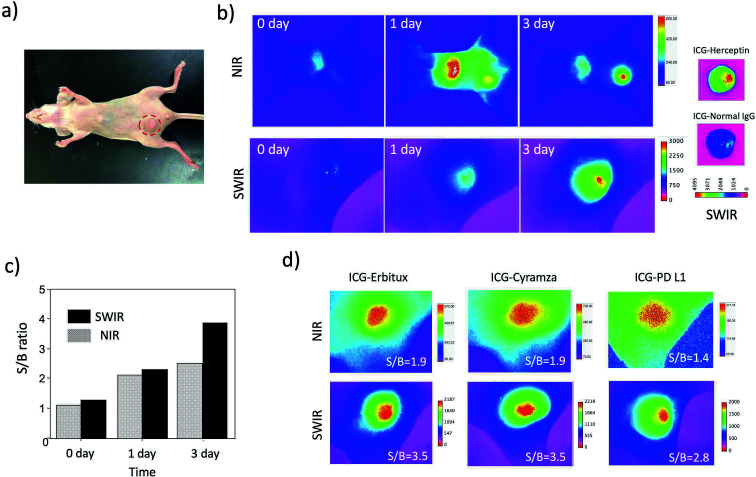
(a) Bright field image of a nude mouse bearing a breast tumour. A red circle shows the position of the breast tumour. (b) NIR (830 nm) and SWIR (>1000 nm) fluorescence images of a nude mouse after the injection of ICG–Herceptin *via* a tail vein. The right images show *ex vivo* SWIR fluorescence images of breast tumours in mice, where the mice were intravenously injected with ICG–Herceptin and ICG–Normal IgG. ICG–Normal IgG was used as negative control. (c) The signal to background (S/B) ratios for the SWIR and NIR fluorescence images (b) of breast tumours. (d) NIR and SWIR fluorescence images of breast tumours taken three days after the injection of ICG–Erbitux, ICG–Cyramza, and ICG–anti-PD-L1 ab. The values of S/B are shown in the inset of each image.

Breast tumour bearing mice were prepared by implanting human breast tumour cell (KPL-4)^31^ to nude mice. The diameter of a tumour was *ca.* 5 mm (Fig. 4a). NIR and SWIR fluorescence images were taken at 0, 1, and 3 days after the injection of ICG–Herceptin to the tail veins of nude mice bearing breast tumours (Fig. 4b). Immediately after the injection of ICG–Herceptin, a strong fluorescence signal of ICG was observed from the liver, while only a weak fluorescence was detected from the breast tumour. At one day post-injection of the ICG–Herceptin, NIR and SWIR fluorescence from the breast tumour was clearly observed. At three days post-injection, most of ICG–Herceptin accumulated to the breast tumour, leading to intense fluorescence from the tumour. To confirm the active accumulation of ICG–Herceptin to the breast tumour, a control experiment using ICG–Normal IgG^33^ was performed. In this case, significant SWIR fluorescence was not observed from the breast tumour compared to the case of an ICG–Herceptin injected mouse (the right images in Fig. 4b).

Comparison of the NIR and SWIR fluorescence images of tumours at three days post-injection showed that the signal to background (S/B) contrast of the SWIR tumour image was 1.5 times higher than that of the NIR tumour image (Fig. 4c). The low background signals in the SWIR fluorescence images (Fig. 4b) resulted from the low tissue-autofluorescence in the SWIR region. When the mouse skin was irradiated by a 785 nm laser (2.5 mW cm^−2^), significant tissue autofluorescence was observed in the NIR region, while almost no autofluorescence was observed in the SWIR region over 1000 nm (Fig. S5†).

Furthermore, we examined the difference in the image contrast of breast tumours stained by other types of molecular imaging probes, ICG–Erbitux, ICG–Cyramza, and ICG–anti-PD-L1 conjugates (Fig. 4d). In all cases, S/B ratios in the SWIR fluorescence images were 1.8–2.0 times better than those in the NIR fluorescence images. This observation was similar with the earlier reported result for the SWIR imaging contrast (50–58%) of brain vasculature and hind-limb vasculature in mice.^7*l*^

### Detection sensitivity of tumours by SWIR fluorescence

**Fig. 5:**
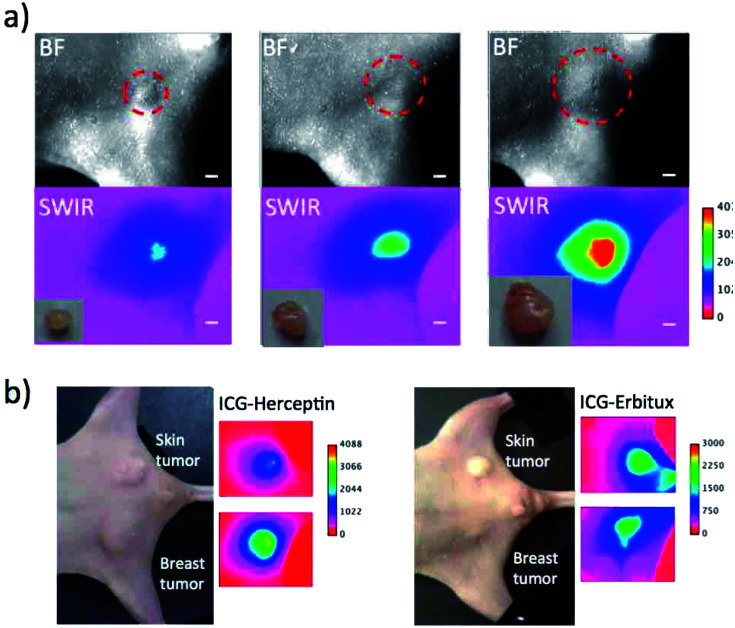
(a) Bright field and SWIR fluorescence (>1000 nm) images for different-sized breast tumours (2 mm to 8 mm in diameter). Images were taken three days after the injection of ICG–Herceptin. The red broken circles show the position of a breast tumour. Inset shows *ex vivo* images of isolated tumours. Scale bar: 1 mm. (b) Bright field and SWIR fluorescence images for the nude mice bearing both breast (KPL-4) and skin tumours (A431). Images were taken three days after the injection of ICG–Herceptin or ICG–Erbitux.

To evaluate the detection sensitivity of tumours by SWIR fluorescence, we conducted SWIR fluorescence imaging for the different-sized breast tumours (*ca.* 2 mm to 8 mm in diameter). SWIR fluorescence images of breast tumours were taken three days after the injection of ICG–Herceptin (Fig. 5a). The intensity of SWIR fluorescence of the breast tumour was linearly increased with increasing the tumour size (Fig. S6†). The small tumor (*ca.* 2 mm in diameter) was clearly detected by SWIR fluorescence. Since tissue autofluorescence in the SWIR region above 1000 nm was very weak, detection sensitivity of breast tumours by SWIR fluorescence was very high enough to be able to detect early-stage cancers which sizes are less than a few millimeters.

Next, we examined the detection sensitivity for the expression level of cancer-specific membrane proteins. SWIR fluorescence imaging was performed for the nude mice bearing both a skin and breast tumour with similar sizes. As shown in Fig. 5b, SWIR fluorescence imaging using ICG–Herceptin showed that the fluorescence intensity of a breast tumour is about 3 times higher than that of a skin tumour (the left image in Fig. 5b). This result indicates the higher expression of HER2 in the breast tumour compared to that in the skin tumour. The SWIR fluorescence imaging using ICG–Erbitux showed that the expression level of EGFR is similar in two different tumours (the right image in Fig. 5b). These observations are consistent with the results of western blotting analysis for the A431 and KPL-4 cells (Fig. 2a). Thus, the SWIR fluorescence imaging using ICG–antibody conjugates has high sensitivity for the detection of the different expression level of membrane proteins in tumours.

### Dual-colour SWIR fluorescence imaging for tumor angiogenesis

Tumours induce blood vessel growth (angiogenesis) by secreting various growth factors (*e.g.* VEGFR) and proteins.^34^ Angiogenesis plays an important role in tumour growth, and the increase in neovasculatures is usually observed with increasing the size of the tumour.^34^ Non-invasive imaging of angiogenesis in tumours is important for early diagnostic and for image-guided management of therapeutic regimens.^35^

**Fig. 6:**
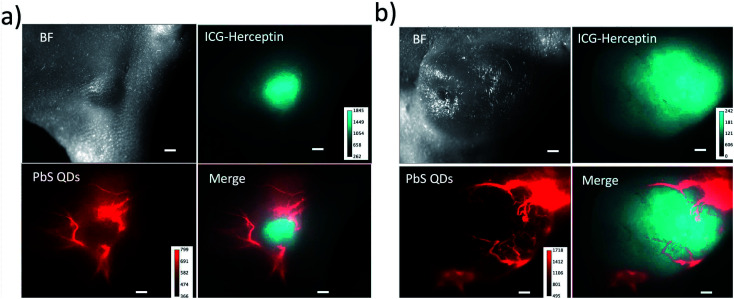
Dual-colour SWIR fluorescence images of breast tumours and neovasculatures in nude mice. The size of tumour is *ca.* 4 mm (a) and 10 mm (b) in diameter. Breast tumours are stained with ICG–Herceptin. The SWIR fluorescence (cyan) from the tumours was detected at the wavelength over 1000 nm by excitation at 785 nm. Neovasculatures were visualized by the injection of glutathione coated PbS QDs through a tail vein of the mouse. The SWIR fluorescence (red) of neovasculatures was detected at 1300 ± 15 nm by excitation at 975 nm. Scale bar: 1 mm.

To perform dual-colour SWIR fluorescence imaging for tumour angiogenesis, we used two types of SWIR fluorescent probe, ICG–Herceptin and PbS quantum dots (QDs).^36^ First, ICG–Herceptin was intravenously injected to a breast-tumour bearing mouse. Three days after the injection of ICG–Herceptin, glutathione-coated water-dispersible PbS QDs (*E*_m_: 1250 nm, Fig. S7†) were intravenously injected to the tumour-bearing mouse. Immediately after the injection of the PbS QDs, dual-colour SWIR fluorescence imaging was performed. By using the SWIR dual fluorescence of ICG (ex:785 nm) and PbS QDs (ex:975 nm), the tumour and neovasculature were separately visualized in the SWIR region (Fig. 6). It should be noted that the neovasculatures in a small-sized tumour (*ca.* 4 mm in diameter) exists at the periphery of a tumour (Fig. 6a), while the neovasculatures in a large-sized tumour (*ca.* 10 mm in diameter) exists both at the periphery and the inside of the tumour (Fig. 6b). These results show that the dual-colour SWIR fluorescence imaging enables the optical detection of tumours as well as tumour angiogenesis.

### SWIR fluorescence imaging of a HER2-positive breast tumour treated with Kadcyla (ado-trastuzumab emtansine)

Finally, we conducted SWIR fluorescence imaging of a breast tumour (KPL-4) treated with Kadcyla (ado-trastuzumab emtansine).^24^ Kadcyla is an antibody-drug conjugate which is used for HER2-positive cancer therapy. Kadcyla comprises anti-HER2 antibody (Herceptin) linked to the anti-mitotic agent emtansine, which is a tubulin polymerisation inhibitor that interferes with mitosis, inducing apoptotic cell death.^37^

**Fig. 7:**
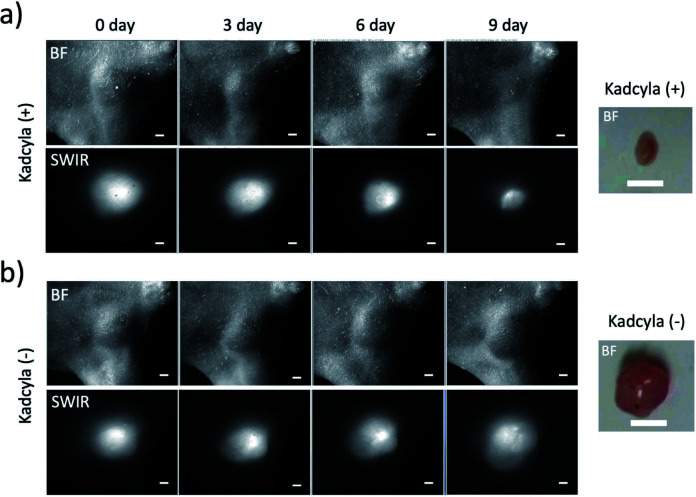
SWIR fluorescence (>1000 nm) images of breast tumours treated with (a) and without (b) Kadcyla (0.2 mg). Kadcyla was intravenously injected to a breast-tumour bearing mouse two days after the injection of ICG–Herceptin. The images were taken 0, 3, 6, and 9 days after the injection of Kadcyla. Scale bar: 1 mm. *Ex vivo* bright field images of breast tumours were taken for the 9 day-mice after the treatment of Kadcyla. Scale bar: 5 mm.

HER2-positive breast tumours were stained by ICG–Herceptin (0.2 mg). SWIR fluorescence of the breast tumour was clearly visualized two days after the injection of ICG–Herceptin, where the size of tumour was about 5 mm in diameter (Fig. 7). In the breast-tumour bearing mouse treated with Kadcyla (0.2 mg), the tumour size significantly decreased (Fig. 7a and S8†): at nine days after the injection of Kadcyla, the tumour size was decreased to be about 2 mm in diameter. This result indicates that the treatment of Kadcyla can induce the apoptosis of HER2-positive breast tumour cells. In contrast, in the mouse without the treatment of Kadcyla, the size a breast tumour was gradually increased (Fig. 7b and S8†). Although Herceptin uses as an anti-cancer drug against HER2-positive breast tumour cells, *in vitro* experiments have shown that Herceptin cannot inhibit proliferation of the HER2-positive KPL-4 cell.^38^

## Conclusion

In this paper, we describe ICG-based SWIR fluorescence molecular imaging for the optical diagnostics of cancerous tumours in mice. We demonstrate that SWIR fluorescence images (>1000 nm) of breast tumours using ICG–antibody conjugates show 1.5–2 times higher S/B contrast compared to the tumour images taken in the NIR region (*ca.* 830 nm). Although the SWIR fluorescence emission of ICG is much weaker than its NIR fluorescence emission, ICG–antibody conjugates has enough sensitivity for the SWIR optical detection of small tumours, which size is less than a few millimetres. Furthermore, we demonstrate that the SWIR fluorescence imaging using ICG–antibody conjugates is useful for the elucidation of the expression level of cancer-specific membrane proteins such as HER2, EGFR, VEGFR-2, and PD-L1 *in vivo*. Our findings show that owing to its high contrast images, ICG-based SWIR fluorescence molecular imaging is highly sensitive compared to conventional ICG-based NIR fluorescence molecular imaging.

We also demonstrate that the SWIR fluorescence imaging using ICG–antibody conjugates enables quantitative analysis of the change in tumour size. Since the fluorescence emission of ICG from the tumours is stable over one month, ICG-based SWIR fluorescence imaging can be used for the evaluation of the effect of anti-cancer drugs. The results of this work show that ICG-based SWIR fluorescence imaging would be useful for the optical detection of cancerous tumors as well as for the optical evaluation of anti-cancer drugs in preclinical research. We believe that ICG-based SWIR fluorescence imaging has potential to use for the optical diagnostics of cancerous tumours (*e.g.* breast tumours) in humans.

## Experimental

### Materials and methods

*N*-Hydroxysuccinimidyl ester derivatives of organic dyes, Alexa488–NHS ester and ICG–NHS ester were purchased from Thermo Fisher Scientific and Goryo Chemicals (Japan), respectively. Heceptin, Cyramza, and Kadcyla were purchased from Chugai Pharmaceutical Co., Ltd (Tokyo, Japan). Erbitux was purchased from Merk Serono. Anti-human PD-L1 antibody was purchased from Bio Cell. Normal human IgG was purchased from FUJIFILM Wako Pure Chemical Corp. (Japan). Glutathione-coated PbS QDs (*E*_m_: 1250 nm) were prepared by the literature method.^36^ Intralipid emulsion (20%) was purchased from Sigma-Aldrich. Breast tumour cells (KPL-4) were kindly provided by Dr Kurebayashi (Kawasaki Medical School). Skin tumour cells (A431) were purchased from RIKEN cell bank. Nude mice (five-week-old female BALB/c nu/nu mice) were purchased from SLC Inc (Japan).

Absorption spectra were recorded with a spectrophotometer (Jasco, V-670). Fluorescence spectra were recorded with a spectrofluorometer (NanoLog, HORIBA, Japan). Fluorescence imaging of cancer cells were performed with a fluorescence microscope (BZ-X700, Keyence, Japan). *In vivo* NIR fluorescence imaging was performed with a fluorescence system (Bruker MS-FX PRO). *In vivo* SWIR fluorescence imaging was performed with a home-built wide-field microscope system.^4*a*^

### Preparation of ICG–antibody conjugates

Alexa488–NHS ester (1 mg) or ICG–NHS ester (1 mg) was resolved to 1 mL of anhydrous dimethyl sulfoxide. To an aqueous solution (0.01 M Na_2_CO_3_) of antibody (Herceptin, Erbitux, Cyramza, and PD-L1), 30 μL of a dimethyl sulfoxide solution of Alexa488–NHS or ICG–NHS was dropwisely added. The coupling reaction was performed for 30 min at room temperature. The dye–antibody conjugates were purified by using a size-exclusion column (PD-10, GE Healthcare) to remove unreacted dyes.

### SDS PAGE of ICG–antibody conjugates

ICG–antibody conjugates were run on a 5–20% polyacrylamide gel (Extra PAGE one Precast gel, Nacalai Tesque) in Tris-glycine-SDS buffer, 200 V for 40 min. NIR fluorescence (*E*_x_: 760 nm/*E*_m_: 830 nm) of ICG was detected first, and then proteins were stained with Coomassie Brilliant Blue (CBB Stain One Super, Nacalai Tesque). Antibody molecules were reduced by dithiothreitol (DTT).

### Fluorescence measurements for ICG–antibody conjugates

NIR excitation spectra and fluorescence spectra of ICG–Herceptin conjugates (1 mg mL^−1^) were measured at the emission of 830 nm and at the excitation of 785 nm, respectively. SWIR fluorescence images of a capillary (1 mm in diameter) filled with ICG–Herceptin conjugates (1 mg mL^−1^) were measured using a solid laser (laser power 2.5 mW cm^−2^) with the excitation of 785 nm. Images are taken by using a long path (>1000 nm) and band path filters (1000 ± 15 nm, 1100 ± 15 nm and 1300 ± 15 nm). NIR fluorescence images of a capillary (*ca.* 0.5 mm in diameter) through an agarose gel (thickness: 0, 1, and 2 mm) were measured with an EMCCD camera (iXon3, Andol) using a band path filter (820 ± 15 nm). The exposure time of the camera was 100 ms. SWIR fluorescence images of a capillary (*ca.* 0.5 mm in diameter) through an agarose gel (thickness: 0, 1, and 2 mm) were measured with an InGaAs CMOS camera (C10633-34, Hamamatsu Photonics). The exposure time of the camera was 30 s.

### Western blotting analysis for cancer cells

The cells were lysed with RIPA buffer (Nacalai Tesque) for 15 min on ice and centrifuged at 10 000×*g* for 10 min at 4 °C to remove insoluble material. Protein concentration was determined by a Protein Assay CBB Solution (Nacalai Tesque). The cell lysates (24 μg) were separated by electrophoresis on 5–20% Extra PAGE One Precast Gel (Nacalai Tesque) at 200 V for 45 min. The protein was transferred to PVDF membrane by a Trans-Blot Turbo Transfer System (BIO-RAD). The membranes were incubated in 5% skim milk for 1 h at room temperature to prevent nonspecific binding. Then, the membranes were incubated in primary antibodies against HER2 (1 : 5000, Santa Cruz Biotechnology), EGFR (1 : 100, Santa Cruz Biotechnology), VEGFR-2 (1 : 200, Santa Cruz Biotechnology), PD-L1 (1 : 500, CUSABIO) or beta-actin (1 : 200, Santa Cruz Biotechnology) for overnight at 4 °C. After washing with TBST, the membranes were incubated with a 1 : 2000 dilution of secondary antibody (goat anti-mouse IgG, HRP conjugate, Millipore) for 1 h at room temperature. After washing with TBST, bands were visualized by treating the membranes with ImmunoStar LD (FUJIFILM) according to manufacturer's instructions and detecting the bioluminescence with LAS-4000 (GE).

### Cellular imaging of cancer cells

KPL-4 and A431 cells were seeded to cell culture dishes (353 001, Falcon 35 mm) and incubated in Dulbecco's Modified Eagle Medium (DMEM) with 10% fetal bovine serum (FBS) for overnight at 37 °C. Alexa488–antibody conjugates were added to the cell and incubated for 10 min at 37 °C. Then, the cells were washed with PBS four times and filled with PBS. Fluorescence images were acquired with a fluorescence microscope (BZ-X700, Keyence Corp., Japan). The excitation and emission filters were 470 ± 20 nm and 525 ± 25 nm, respectively.

### Tumour-bearing nude mice

A suspension of KPL-4 or A431cells (∼10^7^ cells per mouse) was transplanted to the ventral region of 5 week old female BALB/c nu/nu mice. After several weeks, a mouse bearing a tumour less than 10 mm in diameter was selected for imaging. For tumour imaging, two hundred μL of an aqueous solution of ICG–antibody conjugates (1 mg mL^−1^) was injected into a tumour-bearing mouse *via* a tail vein. For the imaging of a breast tumour treated with Kadcyla, Kadcyla (0.2 mg) was intravenously injected to a breast-tumour bearing mouse two days after the injection of ICG–Herceptin. Then, the image of a breast tumour was taken 0, 3, 6, and 9 days after the injection of Kadcyla. All animal procedures were performed in accordance with the Guidelines for Care and Use of Laboratory Animals of RIKEN and approved by the Animal Ethics Committee of RIKEN.

### *In vivo* NIR and SWIR fluorescence imaging

NIR fluorescence images (*E*_x_: 760 nm, *E*_m_: 830 ± 20 nm) of nude mice were taken by using an *in vivo* fluorescence imaging system (Bruker, MS FX PRO). Exposure time of an imaging camera was 30 s. SWIR fluorescence images (*E*_x_: 785 or 975 nm, *E*_m_: >1000 nm) of mice were taken by using a home-built imaging system with an InGaAs CMOS camera (C10633-34, Hamamatsu Photonics) Exposure time was 30 s. A solid laser (laser power 2.5 mW cm^−2^) was used as an excitation light source of 785 nm. SWIR fluorescence images are taken by using a long path filter (>1000 nm).

## Conflicts of interest

There are no conflicts to declare.

## Supplementary Material

RA-010-D0RA04710D-s001
