# Supplementary material for: Shortwave-infrared (SWIR) fluorescence molecular imaging using indocyanine green–antibody conjugates for the optical diagnostics of cancerous tumours
Source: RSC Adv. 2020 Jul 28;10(47):28171–9. doi: 10.1039/d0ra04710d (PMC9055667; doi:10.1039/d0ra04710d)
Supplement: RA-010-D0RA04710D-s001 [file RA-010-D0RA04710D-s001.pdf]

Electronic Supplementary Material (ESI) for RSC Advances.
This journal is © The Royal Society of Chemistry 2020

Electronic Supplementary Information (ESI)

Shortwave-infrared (SWIR) fluorescence molecular imaging using indocyanine green-antibody conjugates for the optical diagnostics of cancerous tumours

Setsuko Tsuboi and Takashi Jin*

RIKEN Center for Biosystems Dynamics Research (BDR), Furuedai 6-2-3, Suita, Osaka 565-0874, Japan. E-mail: tjin@riken.jp

Supporting Figures S1-S8

Fig. S1 Fluorescence spectra of Alexa488-antibody conjugates.

Fig. S2 Fluorescence spectra of ICG-antibody conjugates.

Fig. S3 SDS PAGE of non-reduced and reduced antibodies labeled with ICG-NHS ester.

Fig. S4 Fluorescence images of A431 and KPL-4 cells

Fig. S5 Autofluorescence spectra of a nude-mouse skin.

Fig. S6 SWIR fluorescence intensity for different-seized breast tumors

Fig. S7 Fluorescence spectrum of glutathione-coated PbS QDs

Fig. S8 The time-course of the change in the size of tumor treated with and without Kadcyla.

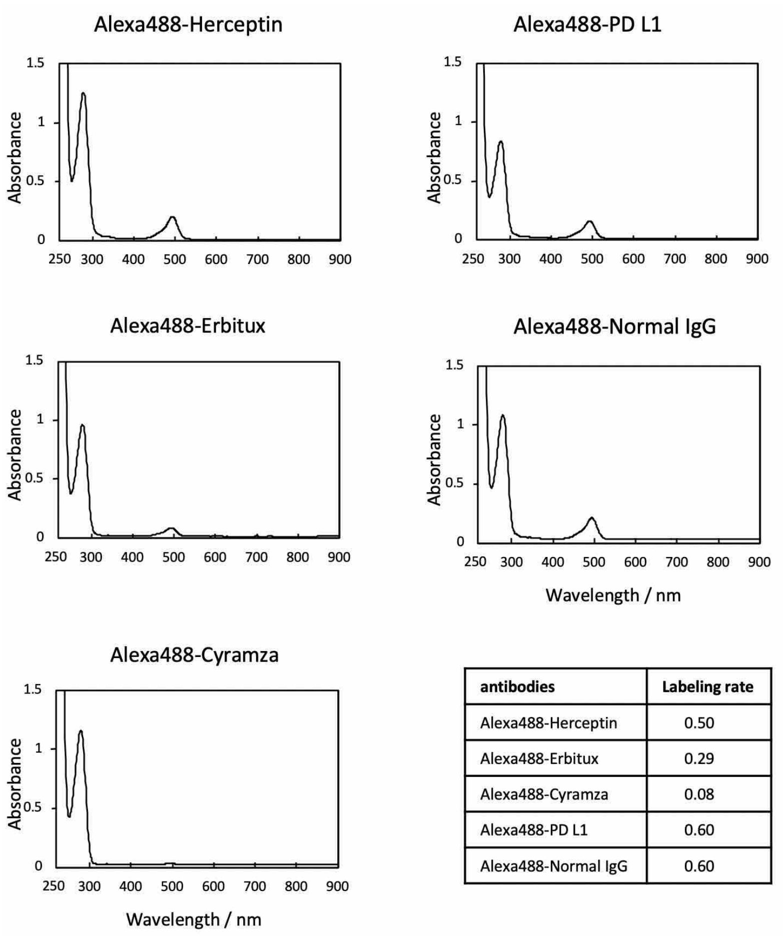

antibodies	Labeling rate
Alexa488-Herceptin	0.50
Alexa488-Erbitux	0.29
Alexa488-Cyramza	0.08
Alexa488-PD L1	0.60
Alexa488-Normal IgG	0.60

Fig. S1 Fluorescence spectra of Alexa488-antibody conjugates. The labeling ratios of dye to antibody were calculated using the extinction coefficients of antibody (216,000 at 280 nm) and Alexa488 (71,000 at 494 nm).

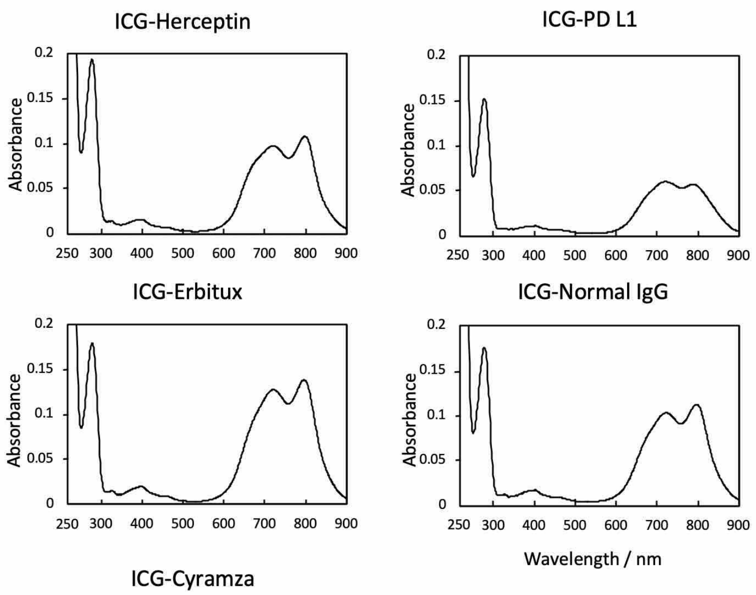

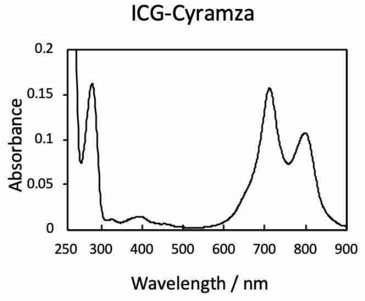

antibodies	Labeling rate
ICG-Herceptin	0.89
ICG-Erbitux	1.21
ICG-Cyramza	1.07
ICG-PD L1	0.56
ICG-Normal IgG	1.00

Fig. S2 Fluorescence spectra of ICG-antibody conjugates. The labeling ratios of dye to antibody were calculated using the extinction coefficients of antibody (216,000 at 280 nm) and ICG (147,000 at 800 nm).

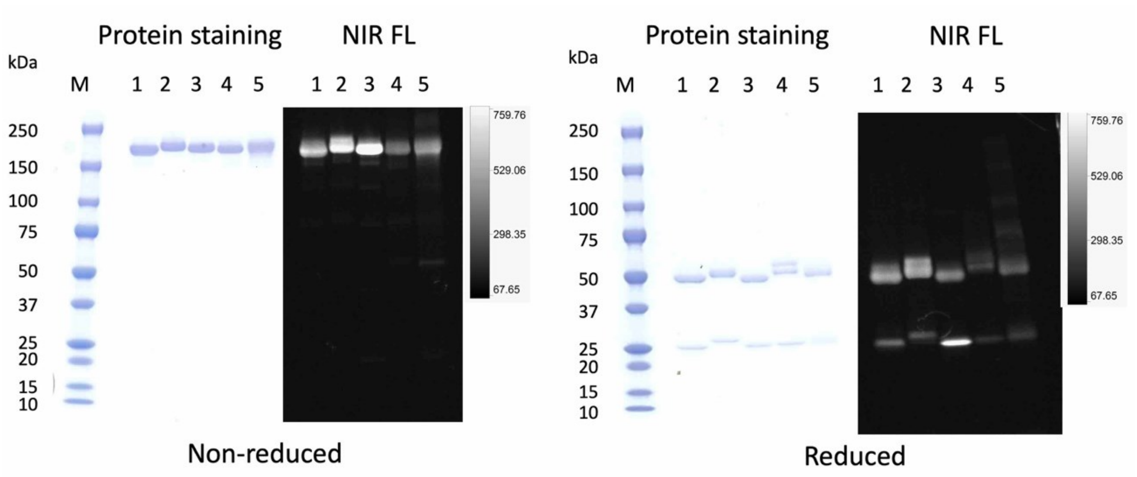

Fig. S3 SDS PAGE of non-reduced and reduced antibodies labeled with ICG-NHS ester. 1: ICG-Herceptin, 2: ICG-Erbitux, 3: ICG-Cyramza, 4: ICG-PD-L1 antibody, and 5: ICG-Normal IgG. Antibody molecules were reduced by dithiothreitol (DTT).

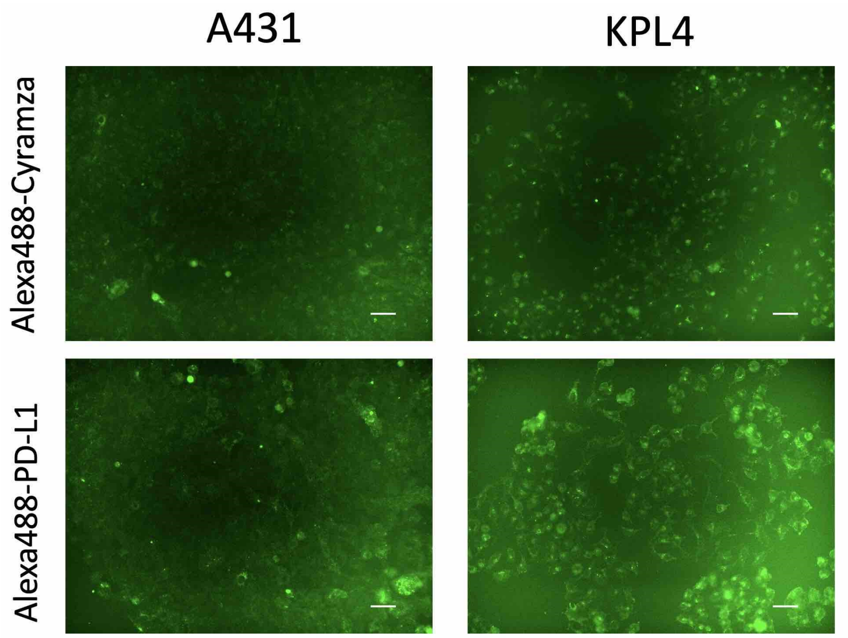

Fig. S4 Fluorescence images of A431 and KPL-4 cells, which were stained with Alexa488-Cyramza and Alexa488-PD-L1. Excitation: 450-490 nm, Emission: 500-550 nm. Scale bar: 50 µm.

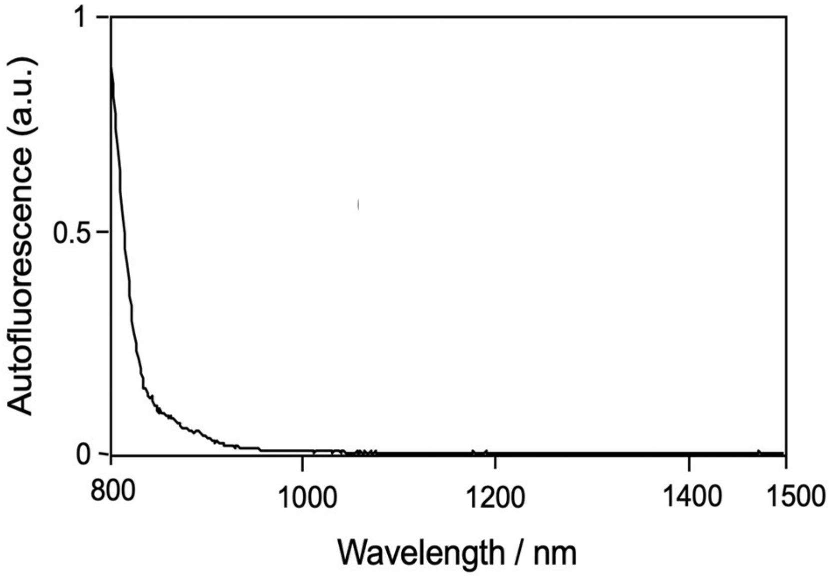

Fig. S5 Autofluorescence spectra of a nude-mouse skin. Autofluorescence was measured by excitation at 785 nm.

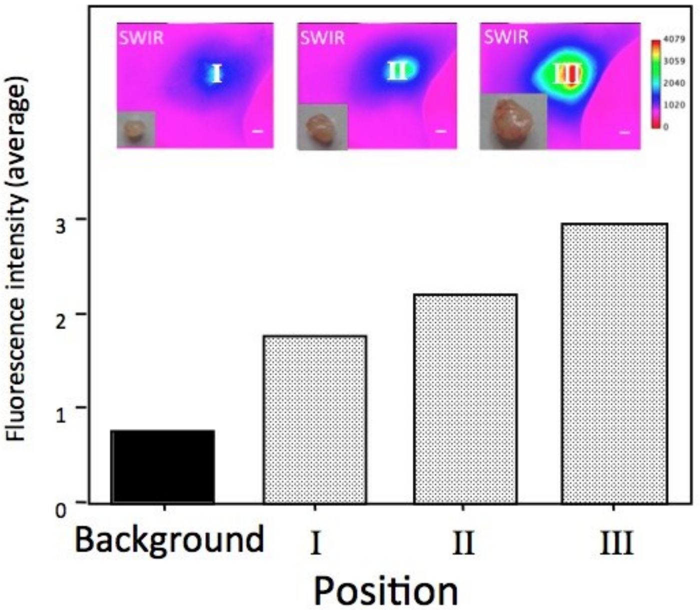

Fig. S6 SWIR fluorescence intensity for different-seized breast tumors from ca. 2 mm to 8 mm in diameter (I, II, and III) and a background signal. ICG-Herceptin was injected to breast tumour bearing mice. Excitation: 785 nm. Emission: >1000 nm. Inset shows the SWIR fluorescence images of the breast tumours.

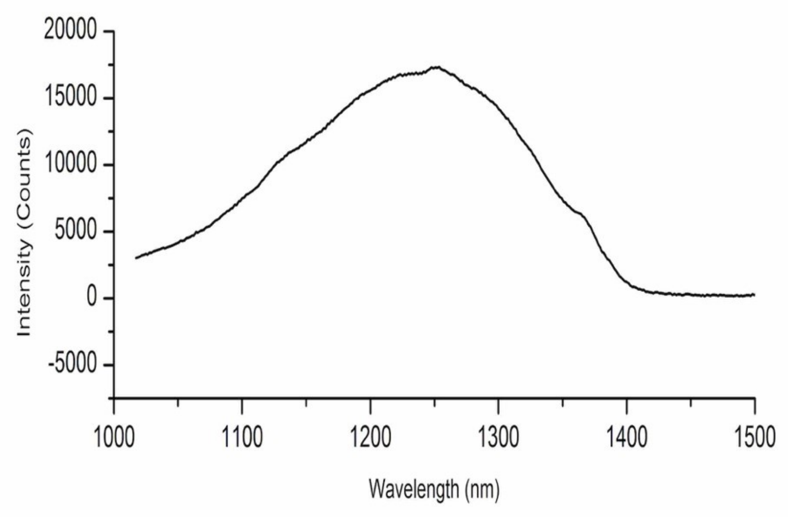

Fig. S7 Fluorescence spectrum of glutathione-coated PbS QDs in water. Excitation: 975 nm.

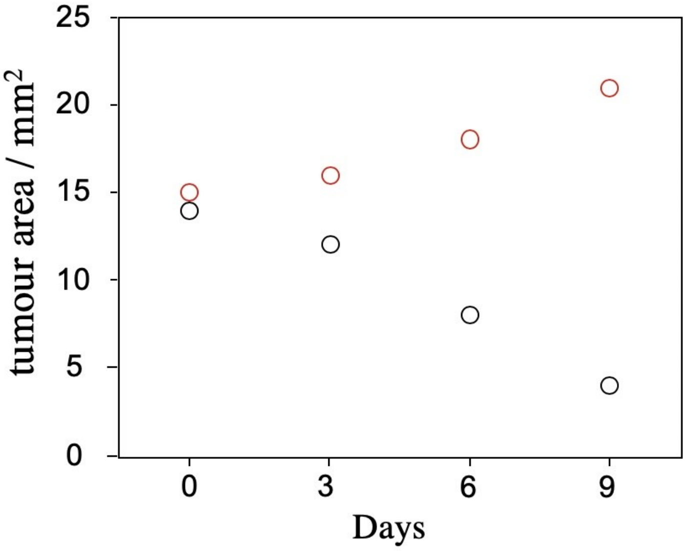

Fig. S8 The time-course of the change in the size of tumor treated with (black circle) and without (red circle) Kadcyla. Kadcyla was intravenously injected to the breast tumour bearing mice two days after the injection of ICG-Herceptin. The area of tumor was measured from its SWIR fluorescence image, which was taken 0, 3, 6, and 9 days after the injection of Kadcyla.
